# Standard versus low-dose computed tomography for assessment of acetabular fracture reduction using novel step and gap measurement technique

**DOI:** 10.1007/s00590-023-03616-9

**Published:** 2023-06-14

**Authors:** Abrianna S. Robles, Amit S. Piple, Donald J. DeSanto, Ashley Lamb, Stephen J. Gibbs, Nathanael D. Heckmann, Geoffrey S. Marecek

**Affiliations:** 1 Department of Orthopaedic Surgery, Cedars-Sinai Medical Center, Los Angeles, CA USA; 2 Department of Orthopaedic Surgery, University of Southern California, Los Angeles, CA USA; 3 Hughston Clinic, Columbus, GA USA

**Keywords:** Posterior wall, Acetabular fractures, Computed tomography, Measurement technique, Gap, Step

## Abstract

**Purpose:**

Quality of reduction is of paramount importance after acetabular fracture and is best assessed on computed tomography (CT). A recently proposed measurement technique for assessment of step and gap displacement is reproducible but has not been validated. The purpose of this study is to validate a well-established measurement technique against known displacements and to determine if it can be used with low dose CT.

**Methods:**

Posterior wall acetabular fractures were created in 8 cadaveric hips and fixed at known step and gap displacements. CT was performed at multiple radiation doses for each hip. Four surgeons measured step and gap displacement for each hip at all doses, and the measurements were compared to known values.

**Results:**

There were no significant differences in measurements across surgeons, and all measurements were found to have positive agreement. Measurement error < 1.5 mm was present in 58% of gap measurements and 46% of step measurements. Only for step measurements at a dose of 120 kVp did we observe a statistically significant measurement error. There was a significant difference in step measurements made by those with greater and those with fewer years in practice.

**Conclusion:**

Our study suggests this technique is valid and accurate across all doses. This is important as it may reduce the amount of radiation exposure for patients with acetabular fractures.

## Introduction

The quality of reduction is a critical factor in the success of acetabular fracture surgery. Anatomic or near-anatomic reduction of posterior acetabular wall fractures results in superior functional and radiographic outcomes following open reduction and internal fixation (ORIF) [1–5]. Conversely, articular malreduction is associated with pain, disability, posttraumatic osteoarthritis and increased risk of reoperation or conversion to total hip arthroplasty [6–8].

Assessment of postoperative reduction is necessary for prognosis and research endeavors. Computed tomography (CT) is superior to plain radiographs when assessing residual displacement following acetabular fracture ORIF, especially in posterior wall fractures [9, 10]. Verbeek et al. [11] proposed a standardized CT-based methodology to assess step and gap displacement with strong interobserver concordance. However, this technique has not been validated against known displacements.

Cumulative radiation dose is high in trauma patients, and there are concerns about increased risk for the development of malignancies, particularly in younger patients [12]. Low-dose CT can reduce radiation dose without sacrificing imaging quality as it relates to the evaluation of fractures in the appendicular skeleton [13–17].

A prior study previously demonstrated that a low-dose CT protocol reduced radiation exposure by 86% compared to the standard-dose protocol without affecting surgeons’ qualitative assessments of acetabular fracture reduction [18]. In this prior study, surgeons were only asked to perform a qualitative assessment of fracture reduction. Verbeek et al. [18] previously validated a quantitative method for assessing gap and step following acetabular ORIF. However, this methodology was assessed only on standard CT scans. It remains to be seen if this well-established quantitative measurement of step and gap can be accurately performed on low-dose CT.

Therefore, the purpose of this study was twofold: (1) to determine if this quantitative measurement technique for step and gap described by Verbeek et al. is accurate when performed on fractures with known displacement and (2) to determine if this methodology can be reliably performed using low-dose CT. We hypothesized that there would be no difference between measured and known displacements across different CT doses.

## Materials and methods

### Dissection and imaging

Eight posterior wall acetabular fractures were created in fresh-frozen cadaveric hips. The fractures were fixed with known combinations of step and gap displacement using standardized internal fixation implants. Metal shims of known thickness were used to displace the fracture and maintained until the fracture was secured. Each specimen was scanned with standard (120 kVp), intermediate (100 kVp) and low-dose (80 kVp) CT protocols. The surgical technique and imaging protocol were previously described [18].

### Review

Four orthopaedic surgeons (two board-certified, fellowship-educated surgeons—an orthopaedic trauma surgeon and an adult reconstruction hip surgeon—and two orthopaedic trauma fellows) measured displacement on all fractures at all doses in two separate sessions at least one month apart. All measurers were blinded to known step and gap displacements, and to CT dose.

### Measurement technique

**Fig. 1:**
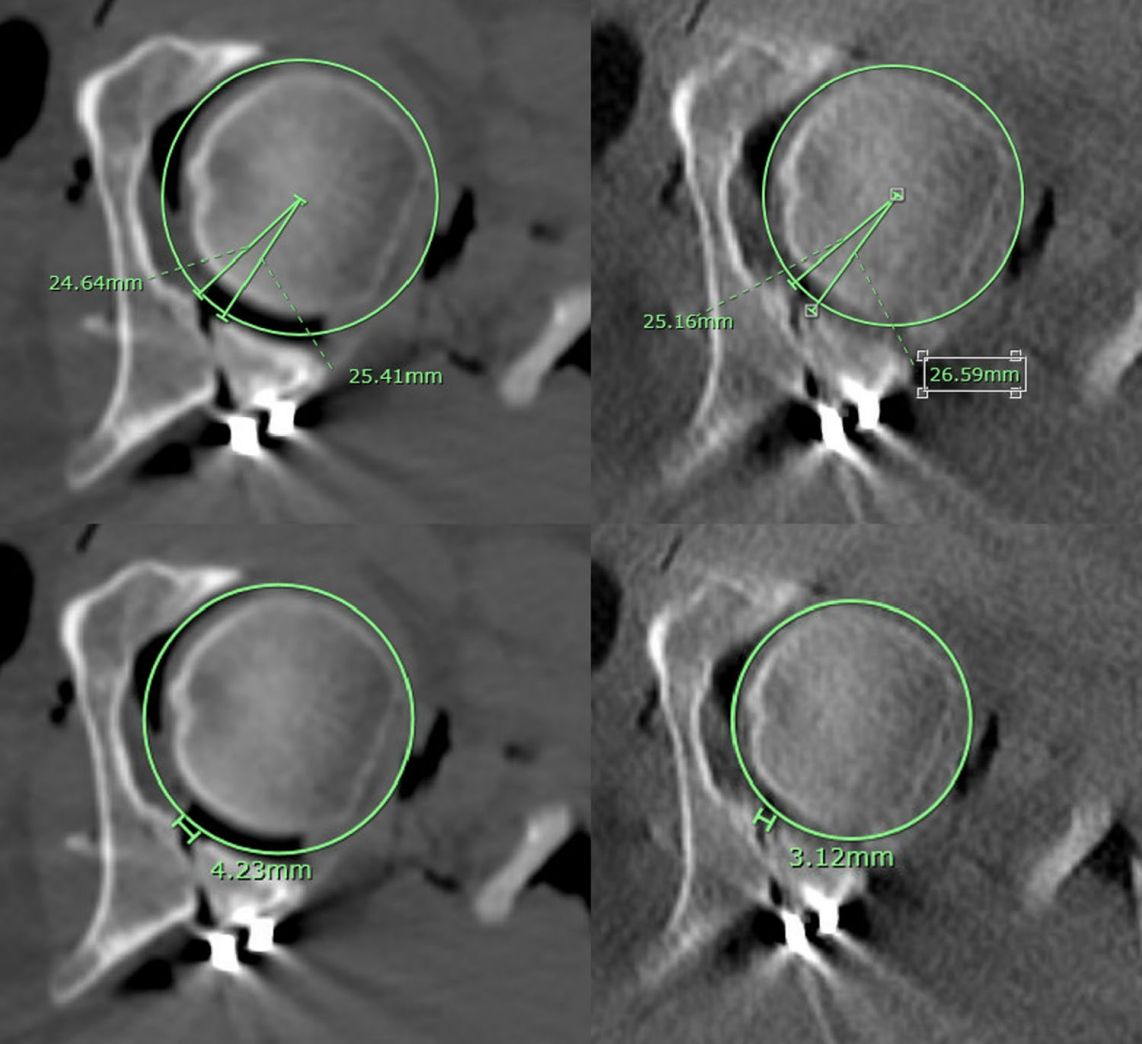
Gap and Step Measurement Technique. **a** gap displacement measurement at 80 kVp, **b** gap displacement measurement at 120 kVp, **c** step displacement measurement at 80 kVp, **d** step displacement measurement at 120 kVp

In each session, measurers were presented with axial, coronal and sagittal cuts of the CT scan and were instructed to select the cut that they felt had the greatest amount of displacement to perform the measurements on. Measurements of step and gap displacement were performed according to the technique described by Verbeek et al. [11], an example of which can be found in Fig. 1. In summary, a best-fit circle was drawn into the acetabulum. Step displacement was defined as the separation of fracture fragments perpendicular to the circumference of the acetabular dome. Step measurements were taken as the largest difference between articular surfaces measured along the radius of the circle. Gap displacement was defined as the area of the acetabular dome where the head is not supported either because of the separation of fracture fragments along the circumference of the dome or because of an area of marginal impaction. Gap was measured as the arc at the circumference.

### Statistics

To test our first hypothesis, the primary outcome was the comparison of the step and gap measurements at 120 kVp to known displacements. At 120 kVP, 8 different patient scans were assessed by 4 raters in 2 different sessions for a total of 64 readings for both step and gap displacement. An average of the 2 sessions’ readings were taken, giving us a new total of 32 readings for both step and gap. This mean value was used to assess the difference in step and gap from known displacements using paired *t*-tests. The *t*-test assessed whether the mean difference of the measurement and known displacement differs from 0. The Bonferroni multiple comparison correction was used to account for multiple independent simultaneous tests being performed (adjusted *p* = 0.002).

To test the second hypothesis of whether measurement error varied by CT dose protocol, the accuracy of the measurement technique was compared across the three different dose levels. We assessed absolute measurements and measurement error—defined as the difference between the known value and the measurement completed by each rater—at 80 kVp, 100 kVp, and 120 kVp. We also calculated the number of step and gap measurements that were statistically different from known values at each dosage level.

Interobserver and intraobserver reliability were evaluated using the intraclass correlation coefficient (ICC) for continuous variables. Intraobserver reliability was assessed by comparing grouped measurements at 80 kVp versus 120 kVp, 100 kVp versus 120 kVp, and 80 kVp versus 100 kVp.

To further assess significant differences that were observed in the step measurement accuracy at the dosing/patient level we performed additional linear mixed models. These models included a random effect for dosing level/patient as well as indicators for dosing and years in practice. For dose level and years in practice, difference in step measurement was used as the outcome. A *p *value less than 0.05 was considered significant. All analyses were performed using R Statistical Software (v3.6.1; R Core 2019, Vienna, Austria).

## Results

At 120 kVP, mean step measurement error was  − 0.76 mm (range  − 4.00 to 2.91 mm, 95% CI  − 1.39 to  − 0.12, *p* = 0.021). Mean gap measurement error was  − 0.27 mm (range  − 3.3 to 3.2 mm, 95% CI  − 0.82 to 0.28, *p* = 0.32). There was no significant difference between measured and known step and gap displacement at 120 kVp individually for each surgeon.

At 100 kVp, mean step measurement error was  − 0.68 mm (range  − 4.44 to 2.91 mm, 95% CI  − 1.45 to 0.09, *p* = 0.081). Mean gap measurement error was  − 0.23 mm (range  − 2.8 to 3.5 mm, 95% CI  − 0.83 to 0.38, *p* = 0.45). There was no significant difference between measured and known step and gap at 100 kVp individually for each surgeon.

At 80 kVp, mean step measurement error was  − 0.27 mm (range  − 4.3 to 3.95 mm, 95% CI  − 1.04 to 0.49, *p* = 0.470). Mean gap measurement error was 0.01 mm (range  − 2.5 to 4.3 mm, 95% CI  − 0.57 to 0.59, *p* = 0.97). There was no significant difference between measured and known step and gap at 80 kVp individually for each surgeon.

Data for average step and gap measurement error by CT dose level can be seen in Table 1, and the number of measurements that were statistically significantly different from the known values can be seen in Table 2.

**Table 1:** Average Step and Gap Measurement Error by CT dose level

	Step				Gap			
	Measurement error	95% CI		*p* value	Measurement error	95% CI		*p* value
80	− 0.27	− 1.04	0.49	0.470	0.01	− 0.57	0.59	0.970
100	− 0.68	− 1.45	0.09	0.081	− 0.23	− 0.83	0.38	0.450
120	− 0.76	− 1.39	− 0.12	0.021	− 0.27	− 0.82	0.28	0.320
Overall	− 0.57	− 0.98	− 0.16	0.006	− 0.16	− 0.49	0.16	0.320

**Table 2:** Statistically Significant* Findings for Step and Gap Measurements at each patient/dosage level

	120	100	80	Total
Gap	0/8	2/8	1/8	3/24
Step	5/8	5/8	3/8	13/24

*Bonferroni multiple comparison correction was used to account for multiple independent simultaneous tests being performed (adjusted *p* = 0.002)

There was no association of step measurement error with radiation dose levels. Measurement error for step was greater for fellows than attendings at every dose level, although this did not reach statistical significance. Measurement error for gap was comparable for fellows and attendings (Table 3).

**Table 3:** Average step and gap measurement error by CT dose level and years of experience

	Step				Gap			
	Measurement error	95% CI		*p* value	Measurement error	95% CI		*p* value
*80*								
Attending	0.13	− 1.14	1.40	0.830	0.16	− 0.80	1.11	0.730
Resident	− 0.67	− 1.63	0.28	0.150	− 0.14	− 0.90	0.63	0.710
*100*								
Attending	− 0.52	− 1.65	0.62	0.350	− 0.29	− 0.99	0.40	0.380
Resident	− 0.84	− 2.02	0.33	0.150	− 0.16	− 1.25	0.92	0.750
*120*								
Attending	− 0.74	− 1.72	0.23	0.120	− 0.34	− 1.14	0.46	0.380
Resident	− 0.77	− 1.71	0.16	0.099	− 0.20	− 1.05	0.65	0.630

Cumulatively (using mean of all 4 surgeon measurements at both sessions), 5 of 8 (62.5%) step measurements at 120kVp, 5 of 8 (62.5%) at 100 kVp, and 3 of 8 (37.5%) at 80 kVp were different than known values. This made a total of 13 out of 24 (54%) step measurements that were significantly different from known values (adjusted *p* < 0.002). Measurement error < 1 mm was present in 6 out of 24 (25%) of samples. Measurement error < 1.5 mm was present in 11 out of 24 (46%) of samples.

For gap measurement, 0 out of 8 (0%) measurements at 120 kVp, 2 out of 8 (25%) at 100 kVp and 1 out of 8 (12.5%) at 80 kVp, were different than known values. This made a total of 3 out of 24 (12.5%) gap measurements that were significantly different from known values (Bonferroni multiple comparison correction adjusted *p* < 0.002). Measurement error < 1 mm was present in 9 out of 24 (38%) of samples, and measurement error < 1.5 mm was present in 14 out of 24 (58%) of samples.

All ICC and Light’s Kappa coefficients were positive except for one, indicating agreement among all raters. Although positive, agreement was lowest in rating of gap displacement, with all Light’s Kappa coefficient values < 0.1. Surgeons with more experience had more accurate ratings (overall percentage correct = 44.8%) than their junior counterparts (overall percentage correct = 41.7%).

The ICC for each dosage comparison can be seen in Table 4. Overall, 80 versus 120 had the lowest level of agreement, and 100 versus 120 had the highest level of agreement. There was no disagreement in either the continuous measurements or categorical variables across the board.

**Table 4:** Interrelator reliability

	Overall	80	100	120
Gap (mm)	0.148	0.120	0.194	0.147
Gap displacement	0.021	− 0.005	0.073	0.007
Step (mm)	0.266	0.120	0.311	0.383
Step displacement	0.135	0.095	0.187	0.095
Overall reduction	0.078	0.027	0.069	0.126

## Discussion

Postoperative CT after acetabular fracture surgery is used in several ways, though how widespread the practice is remains unknown. Evaluation of the surgical result including the quality of the fracture reduction, the presence of retained loose osteochondral fragments or malpositioned implants is likely the most common use. Postoperative CT can identify the need for reoperation, although this is uncommon and indications are surgeon-dependent [19–21]. Orthopaedic trauma surgeons are performing fewer pelvic and acetabular fracture surgeries per surgeon over time [22]. Given this decreased surgical volume and the unchanged, high learning curve of acetabular fracture surgeries, postoperative CT scan can potentially provide a wealth of educational value to the developing surgeon. Lastly, the CT scan can glean higher quality information on the quality of reduction that can be used for prognosis or other academic purposes such as comparing surgical approaches or fixation strategies. It is the latter with which the evaluation method proposed by Verbeek et al. is concerned.

Previous studies have established a strong positive correlation between reduction quality and hip survivorship [18, 23–25]. In a series of 211 patients with acetabular fractures, Verbeek et al. found that only 10% of the 99 patients with adequate reductions on postoperative CT underwent conversion to total hip arthroplasty compared to 36% of those with inadequate reductions [9]. A follow-up study described a novel CT-based technique for measuring step and gap displacement and demonstrated that using this standardized method for measurement resulted in improved interobserver agreement [11]. The present study builds off this previous work in two ways: first, by validating the measurement method against known, experimentally set displacement values; and second, by showing that the measurement method may be used with a low-dose CT protocol without affecting accuracy.

The step and gap measurement technique proposed by Verbeek et al., [11] is reproducible with standard dose (120 kVp) CT scans. The interobserver reliability was high between surgeons, suggesting that even with some instructions left to surgeon discretion (e.g., “area of maximal displacement”) the measurement technique held up well. The technique also performed well for gap displacement, as none of the mean cumulative measurements were different from the known. However, when measuring step displacement there was substantial error with nearly 63% of measurements different from known values at standard dose. This suggests that the technique may be precise but not accurate.

The accuracy of measurement was not affected by the CT dose protocol, suggesting that this measurement technique can be used equally well with low dose CT scans. Both measurement error and percent of accurate measurements remained consistent across doses. Interestingly, measurement of gap was best at higher doses, while measurement of step was worse at higher doses, though not significantly so.

Several reasons may explain why measuring step displacement using this technique produced greater error. When presented with axial, sagittal, and coronal imaging, measurers were asked to choose the view for measurement technique based on which one seemed to have the greatest amount of step or gap; as such, surgeons may have selected different images. More actions were required when conducting step displacement measurement versus gap measurement. To measure step displacement, measurers were instructed to place a best-fit circle in the acetabulum, draw two radii connecting to the high and low end of the step, and then find the difference in lengths. Inaccuracy in any measurement could result in error, as opposed to the one line needed to assess gap. The PACS system was not familiar to all surgeons, which may have led to an adjustment period while figuring out how to best utilize the software. Lastly, due to the method for creating displacement, it is possible that slight variations in step existed along the curved articular surface when displacement was created with a straight metal shim. Conversely, gap measurements would be uniform along the plane of the fracture using this technique.

Attending surgeons were more accurate in measurement of step than fellows (although not significantly so), however, for gap measurement, measurement error was about the same. In relation to the significant error for step measurement, it is possible that measurers with decreased years in practice may be less likely to perform the technique correctly. This may also be in part due to reasons previously discussed, such as unfamiliarity with PACS system, surgeon discretion with anatomic selection, and increase in number of steps to complete the technique.

There are several limitations of this study. First, this was a cadaveric study which may not accurately reflect in vivo conditions. Although attempts were made to mimic these conditions as best as possible by repairing soft tissues, etc., air was introduced into the joint space which produced artifact in some cases. Second, we utilized a posterior wall model, which may limit the generalizability to other types of acetabular fractures. Additionally, the small number of measurements and sessions limited the number of data points to analyze. Lastly, as mentioned previously, the displacements were created using a metal shim but were not re-measured prior to scanning, which may have led to under-estimation of the displacement (i.e., an intended 5 mm gap was actually 5.1 mm).

The results of this study confirm that the digital measurement technique proposed by Verbeek et al. [11] is reproducible and is accurate for gap displacement. However, some error is present when measuring step displacement, which is more critical for prognosis (cite Verbeek). The technique is also reproducible across various CT doses, meaning that a *quantitative* assessment can be done at low doses, instead of just a *qualitative* assessment as previously suggested by Gibbs et al. [26].

The use of low dose CT can markedly decrease the amount of radiation to which orthopaedic patients with acetabular fractures are exposed, leading to decreased risk of potential malignancy [12]. Compared to 120 kVp CT scans, 100 kVp and 80 kVp represent a dose reduction of 47.7% and 86.7% respectively. As radiation exposure is best understood measured as effective dose (millisieverts, or mSv), previous literature has defined the exposure from a standard dose CT scan as 3.17 mSv and for the low-dose CT scan as 0.42 mSv. Equated to standard chest X-rays (0.08 mSv), the standard CT dose exposes the patient to the same amount of radiation as 40 chest X-rays, while the low-dose CT scan exposes the patient to the same amount as 5 chest X-rays [26]. This may enhance acceptance of the use of postoperative CTs, which would allow scientists to better balance acquiring academic knowledge with patient safety.

This standardized quantitative CT-based technique for measurement of acetabular fracture reduction resulted in reproducible measurements of step and gap and accurate measurements of gap displacement in a posterior wall fracture model. The measurements were consistent among surgeons at different levels of training, and measurements were not negatively affected by the use of a low-dose CT protocol. Future studies may wish to evaluate the reproducibility of these measurements on intraoperative three-dimensional fluoroscopy-based scans.
